# Rams Have Heart, a Mobile App Tracking Activity and Fruit and Vegetable Consumption to Support the Cardiovascular Health of College Students: Development and Usability Study

**DOI:** 10.2196/15156

**Published:** 2020-08-05

**Authors:** Michelle C Krzyzanowski, Paul N Kizakevich, Vanessa Duren-Winfield, Randall Eckhoff, Joel Hampton, Loneke T Blackman Carr, Georgia McCauley, Kristina B Roberson, Elijah O Onsomu, John Williams, Amanda Alise Price

**Affiliations:** 1 RTI International Research Triangle Park, NC United States; 2 Winston-Salem State University Winston-Salem, NC United States; 3 University of Connecticut Storrs, CT United States

**Keywords:** exercise, cardiovascular disease, diary, diet, mHealth, mobile phone

## Abstract

**Background:**

With the increasing use of mobile devices to access the internet and as the main computing system of apps, there is a growing market for mobile health apps to provide self-care advice. Their effectiveness with regard to diet and fitness tracking, for example, needs to be examined. The majority of American adults fail to meet daily recommendations for healthy behavior. Testing user engagement with an app in a controlled environment can provide insight into what is effective and not effective in an app focused on improving diet and exercise.

**Objective:**

We developed Rams Have Heart, a mobile app, to support a cardiovascular disease (CVD) intervention course. The app tracks healthy behaviors, including fruit and vegetable consumption and physical activity, throughout the day. This paper aimed to present its functionality and evaluated adherence among the African American college student population.

**Methods:**

We developed the app using the Personal Health Informatics and Intervention Toolkit, a software framework. Rams Have Heart integrates self-reported health screening with health education, diary tracking, and user feedback modules to acquire data and assess progress. The parent study, conducted at a historically black college and university-designated institution in southeastern United States, consisted of a semester-long intervention administered as an academic course in the fall, for 3 consecutive years. Changes were made after the cohort 1 pilot study, so results only include cohorts 2 and 3, comprising a total of 115 students (n=55 intervention participants and n=54 control participants) aged from 17 to 24 years. Data collected over the study period were transferred using the secure Hypertext Transfer Protocol Secure protocol and stored in a secure Structured Query Language server database accessible only to authorized persons. SAS software was used to analyze the overall app usage and the specific results collected.

**Results:**

Of the 55 students in the intervention group, 27 (49%) students in cohort 2 and 25 (45%) in cohort 3 used the Rams Have Heart app at least once. Over the course of the fall semester, app participation dropped off gradually until exam week when most students no longer participated. The average fruit and vegetable intake increased slightly, and activity levels decreased over the study period.

**Conclusions:**

Rams Have Heart was developed to allow daily tracking of fruit and vegetable intake and physical activity to support a CVD risk intervention for a student demographic susceptible to obesity, heart disease, and type 2 diabetes. We conducted an analysis of app usage, function, and user results. Although a mobile app provides privacy and flexibility for user participation in a research study, Rams Have Heart did not improve compliance or user outcomes. Health-oriented research studies relying on apps in support of user goals need further evaluation.

## Introduction

### Background

By 2025, nearly three-fourth of all internet users will access the web exclusively by mobile phone. Young people, in particular, show heavy usage: 53% own a smartphone by 11 years of age, and 84% of teenagers own a smartphone [1]. This increasing reliance on mobile phones has sparked the creation of self-help health apps, known as mobile health (mHealth) [2]. They aim to improve public health by supporting patient-led self-care [3,4]. They provide interactive, educational modules, and around 4 million versions can be readily downloaded through both the Google Play and Apple platforms [5]. They can be used for fitness tracking to address obesity, journal writing to address diabetes or mental health, and tracking other areas of concern, such as the menstrual cycle [6,7]. App developers see a growing youth market.

A popular mHealth focus is on fitness and diet tracking [6,7]. Fewer than 18% of American adults meet the daily recommendations for fruit and vegetable servings, and only one-third engage in the recommended amount of physical activity each week [8]. Although the transition from adolescence to young adulthood can be crucial in shaping weight-related behavior and lasting health patterns [9], only 10% of young adults entering college (18 to 30 years of age) are likely to adhere to dietary guidelines [10]. Studies also report a significant decrease in physical activity and an increase in sedentary behavior in this period [11]. Methods to address these health concerns include the promotion of healthy nutritional and dietary habits and physical activity [12-14].

The risk of obesity and high blood pressure (BP) is especially pronounced in minorities, leading to negative health outcomes when not detected early [15]. The incidence rate of diabetes among African Americans (AAs) is approximately twice that among non-Hispanic whites. Risk factors for cardiovascular disease (CVD) in college students include obesity and physical inactivity, which are disproportionately higher among AAs [16,17], and studies have shown that AA college students do not consistently engage in physical activity [18,19]. In a study conducted at a historically black college and university (HBCU), average BP was higher among AA students than their non-Hispanic white counterparts [20]. Whereas studies of the obesity epidemic have increased in recent years, few target AA college students.

To address this gap, we used the Personal Health Informatics and Intervention Toolkit (PHIT) framework to support a CVD risk factor intervention. mHealth apps have demonstrated success in improving medication adherence and supporting mental health interventions [21-23]. In particular, self-care methods have shown promising results in helping with depression and anxiety and encouraging a healthier lifestyle [24,25]. The Rams Have Heart mobile app targets healthy behaviors, tracking fruit and vegetable consumption, and physical activity throughout the day. It was not designed to actively support behavior change *per se* but to monitor the adaptation of concepts from the CVD-oriented college course. This study aimed to present the functionality of the Rams Have Heart mobile app and evaluated its usability and adherence in a vulnerable student population.

## Methods

### Ethics Approval

This study was approved by the Winston-Salem State University institutional review board (IRB). In addition, the Research Triangle International (RTI) Committee for the Protection of Human Subjects approved the RTI’s role in the analysis of deidentified data.

### Study Design and Population

This secondary analysis aimed to evaluate the usability and acceptability of the Rams Have Heart app, a tool developed as part of a larger pilot study to test an evidence-based CVD intervention in a susceptible demographic-AA college students.

This study was conducted at an HBCU in southeastern United States, where 73.6% of the student body is AA [20]. After the university’s IRB reviewed and approved the study, the IRB at the supporting contracting institution approved its study personnel’s access to the deidentified data for analysis. The CVD course was made available to students during the fall semester for 3 consecutive years; participants in each iteration are referred to as cohorts 1, 2, and 3. Cohort 1 was essentially in a pilot study, as the course and app were evolving, and a variety of revisions were made before starting cohort 2. Therefore, the study population, analyses, and results are limited to cohorts 2 and 3.

A total of 109 students participated across cohorts 2 and 3. Of these, 55 (50.4%) were assigned to the intervention group, who took the CVD risk-reduction course and were directed to use the Rams Have Heart app, and 54 (49.5%) were assigned to the control group, who took the traditional health course and did not use the app. All were full-time students at the institution, aged from 17 to 24 years. Cohort 2 was under study from fall 2017 to spring 2018, and cohort 3, from fall 2018 to spring 2019. The main course activity took place during the fall semester.

### App Development

#### Personal Health Informatics and Intervention Toolkit

Rams Have Heart was developed using PHIT, a software-development framework geared toward research-oriented mobile apps [21,22]. PHIT apps comprise 5 primary elements: (1) user interactions involving forms, diaries, cognitive tasks, and games; (2) optional sensor data acquisition and processing; user-interaction scheduling, notification, and management; and data visualization; (3) configuration and control scripts, virtual advisor processing, and activity and intervention logic; (4) database, privacy, and security support; and (5) study assets, such as educational materials and audiovisual media.

PHIT interactive instrument modules and configuration settings were implemented using extensible markup language and a custom language called PHITScript to construct program logic and activate such app functions as dynamically altering menus, controlling data collection, or scheduling notifications. The framework runtime loaded such modules and settings to render the mobile app dynamically tailored to the user and adaptable to the study protocol. The architecture has contributed reusable data collection and intervention modules to a growing library for mobile app studies.

Apps using the PHIT framework run on the user’s device without an active internet connection. Raw and derived data are tagged with the protocol, participant, and other contextual information, encrypted, and stored locally in the app space. Whenever the internet is available, data are uploaded to a study-specific server via the secure https protocol. Individual and aggregate data can then be off-loaded via a password-protected portal for review and analysis. PHIT is based on Apache Flex and Adobe Integrated Runtime (AIR) technology, both open source and widely used for mobile game development. By using Apache Flex AIR, PHIT-based apps executed on both Android and iOS devices perform on smartphones and tablets and can be adapted for desktop platforms.

### Rams Have Heart Mobile App

#### App Startup and BMI Calculation

**Figure 1:**
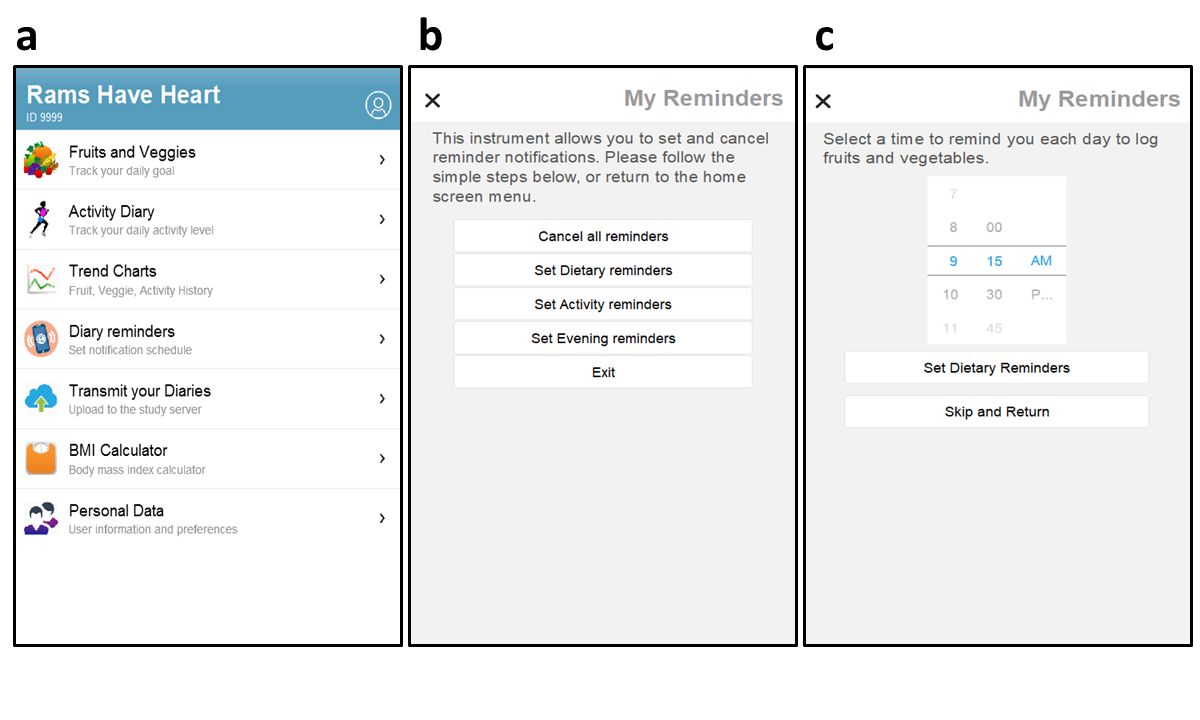
Home screen menu for the Rams Have Heart app (a) and screens for selecting (b) and setting (b) daily diary reminders.

Rams Have Heart integrated health screening with dietary and exercise education, food consumption and activity tracking, and graphic feedback showing progress. A simple, user-friendly home screen menu (Figure 1) listed app functions—tracking diaries, health information, personal feedback, and support modules—with an illustrative icon, a function label, and a brief descriptive statement to aid understanding.

When first running the app, the study participants entered their assigned identifier (ID) twice for verification. It was displayed on the blue home screen header above the function list for easy reference. All data records were tagged with this ID, and no personally identifiable information (PII) was saved. The research investigators maintained a study roster linking the ID to student contact information, and any other PII (offline and separate from the app), and any app-acquired data.

Next, participants were asked to set a time when notifications reminding them to keep up with dietary, activity, and evening diary entries would be posted (Figure 1). The *daily reminders* function was also listed on the home screen menu, allowing users to cancel or reschedule them to suit their needs.

**Figure 2:**
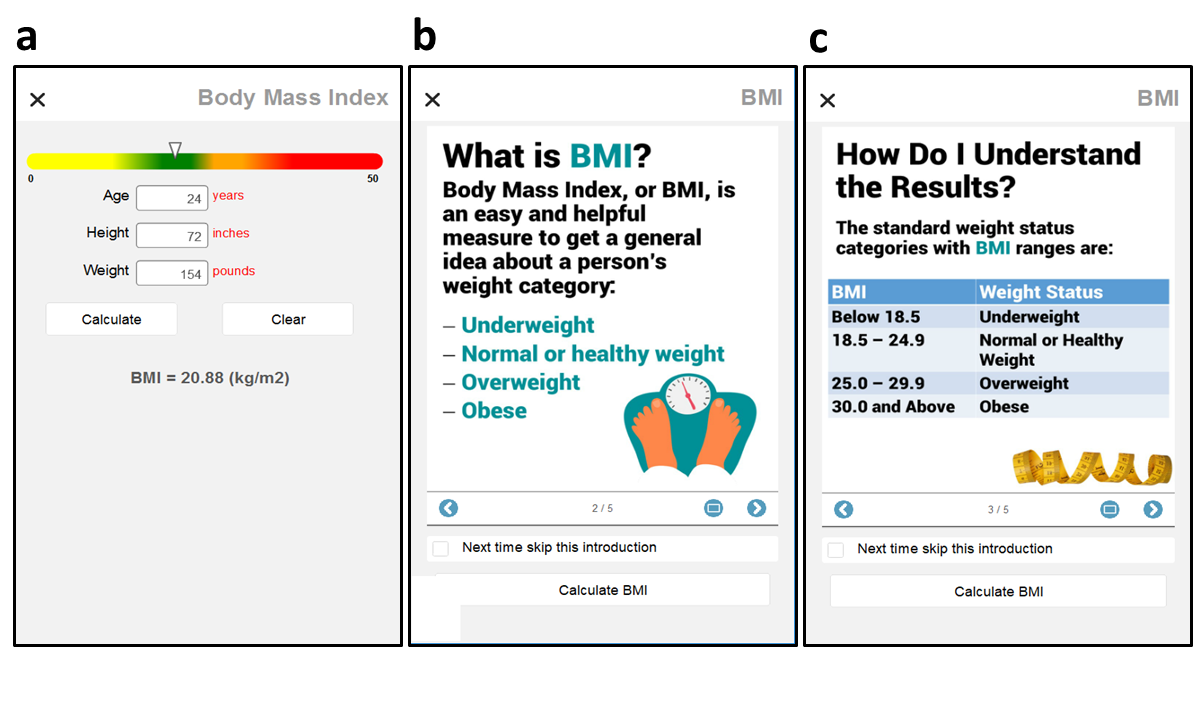
BMI data entry and calculator (a), along with sample screens (b, c) from the five-slide set of BMI educational materials.

The final startup task was to enter self-reported personal data (ie, sex, age, height, weight) and save them for subsequent BMI calculations. Participants then needed only to enter their weight periodically to recalculate BMI and observe progress (Figure 2). Should any other personal data change (eg, age) during the study, it could be re-entered via the home screen menu. To support participant understanding, a series of graphic materials on BMI and its interpretation were available for reference (Figure 2).

#### Dietary and Physical Activity Diaries

**Figure 3:**
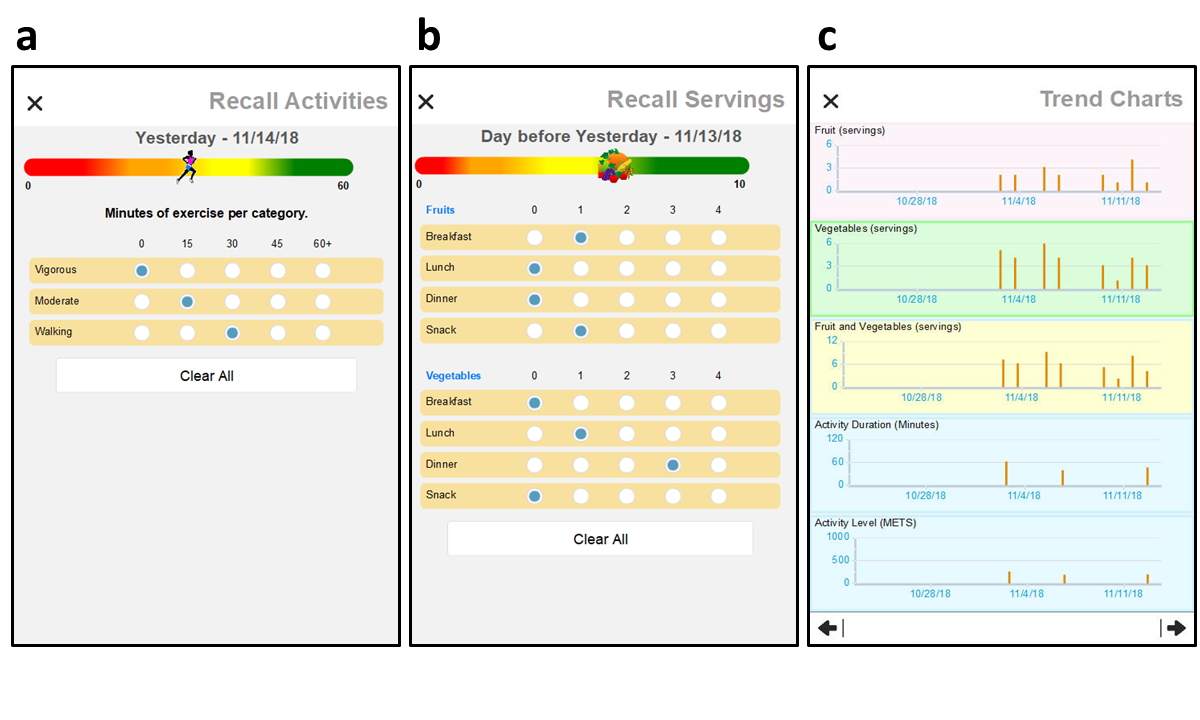
Example diary entry screens for daily tracking of fruit & vegetable consumption (a) and physical activity (b). Trend charts provide feedback on behavior goals over three weeks of recent entries (c).

The primary function of the app was to support the daily recording of fruit and vegetable consumption and physical activity. The fruit and vegetable diary provided simple tap entry for up to four servings at each meal or when snacking across the day (Figure 3) for a range of 0 to 32 servings of fruits and vegetables per day. Servings data could be entered throughout the day or by recall at the end of the day and revised any time during the day. At the top of the screen, a color-coded progress bar advances toward a maximum of 10 servings. The green zone indicates whenever participants achieved at least six total servings, with a yellow transition to the 4 to 6 serving range. A graphic cluster of fruits and vegetables marks progress toward meeting the daily goal of 6 fruit or vegetable servings.

The physical activity diary used a similarly simple design, providing 5 levels of vigorous activity, moderate activity, and walking (Figure 3) across each day. The concept was modeled on the International Physical Activity Questionnaire (IPAQ). Participants could enter these data immediately after completing the activities or by recall later in the day and update or edit them at any time on the current day. At the top of the screen, a color-coded progress bar shows the cumulative weighted minutes of physical activity across the day. Weighted minutes were calculated using an IPAQ-adapted formula as follows:

Weighted minutes = (2.0 × vigorous minutes) + moderate minutes + (0.5 × walking minutes)

As new entries were made, the progress indicator advanced toward a maximum of 60 weighted min. The green zone indicated whenever participants achieved at least 45 weighted min, with a yellow transition for at least 30 weighted min. A graphic icon of a runner was used to indicate cumulative effort compliance.

Diary trend charts provided feedback to help students meet personal goals throughout the study intervention (Figure 3). A concise single screen presented a 3-week history of diary entries, allowing users to reflect on their eating and physical activity behaviors. This approach also aimed to support behavior change and encourage compliance with a daily diary entry.

**Figure 4:**
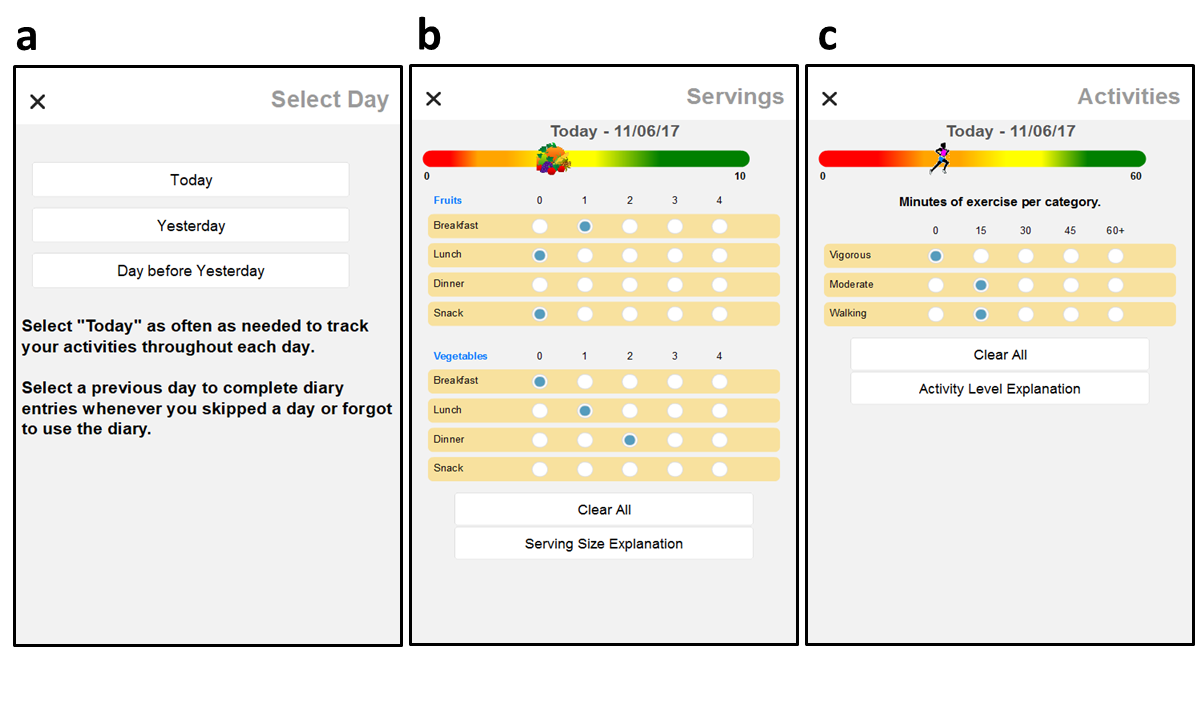
Users may catch up on missed diary entries for one or two days behind schedule (a). Labeling is changed to remind users that recall data are to be entered for specified day (b, c).

During the first year of our study, participants frequently did not provide diary data every day. As they may have simply forgotten to make entries on a certain day, we revised the app for cohorts 2 and 3 to allow them to *catch up.* They could now fill in data for days they missed or forgot to enter for up to two days via recall. We added a form that appeared when participants entered either diary, asking whether the next entries will record (1) the current day or (2) 1 day or (3) 2 days earlier (Figure 4). Once the day was selected, either the fruit and vegetable dairy (Figure 4) or the physical activity diary (Figure 4) was presented. Labeling at the top of the diary screens was changed to remind the participant that recall data were to be entered for the specified day.

**Figure 5:**
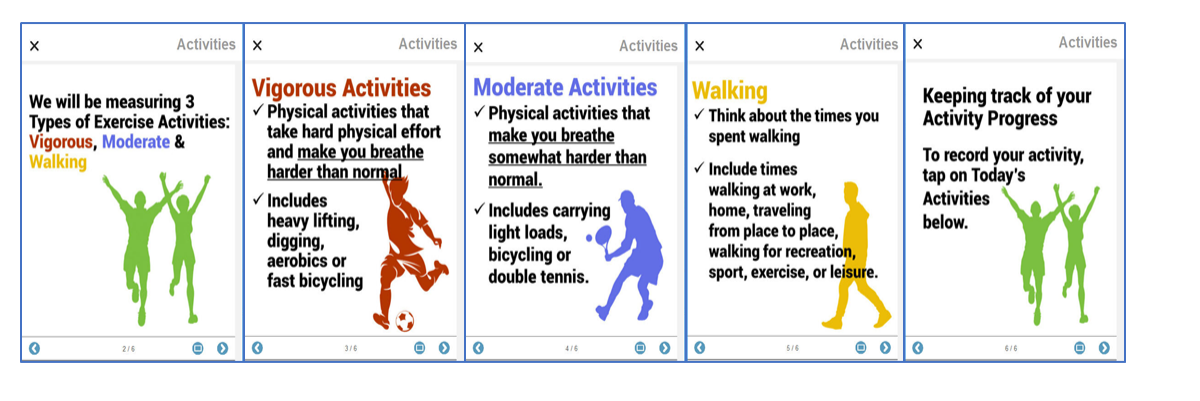
Explanation of levels of activity effort to support quality diary entries.

**Figure 6:**
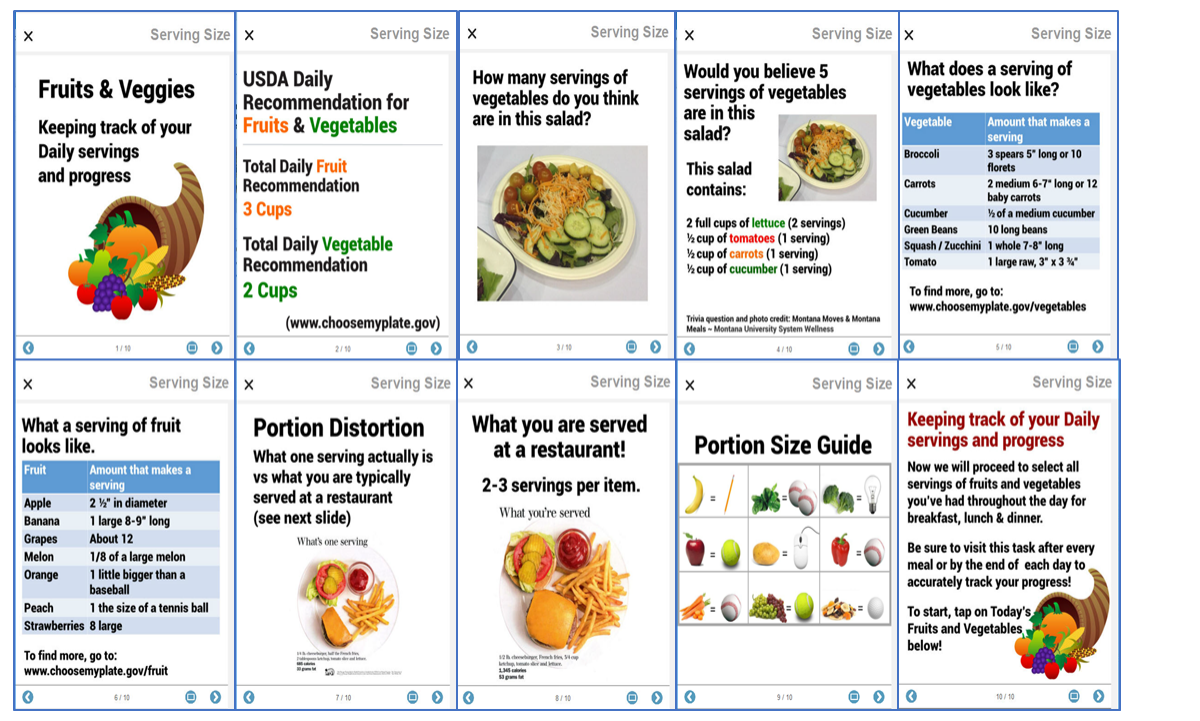
Explanation of fruit and vegetable serving size to support quality diary entries, along with educational materials supporting healthy eating choices and behaviors.

The first-time participants entered either the diary module, a series of slides would explain how to fill in the ensuing diary entries and reinforce the educational content and its relationship to the CVD risk–reduction course (Figures 5 and 6). Once reviewed, they could be disabled for the participants’ future data entry, but they were always available as reference materials via a simple button tap.

#### Data Storage, Privacy, and Security

Participants were provided with a unique ID to link all acquired data to that individual. They also entered a self-defined, secret, 4-digit Personal Identification Number (PIN) to prevent access by other individuals. When the app is in use, its screen will deactivate after a set period (eg, 2 min) of no interaction, and a screen cover displayed to hide current data or activity. Once deactivated, the secret 4-digit PIN must be entered to unlock the screen and allow the participant to continue.

All acquired and calculated data were stored locally in an encrypted Structured Query Language (SQL)ite database within the mobile app. Data reflecting app function, such as date-entry time stamps and the length of time spent entering diary information, were also saved to the secure in-app database. Each time the users exited a diary, they were offered the opportunity to upload the acquired data to the secure central study data server or to defer it. The app then returned the user to the home screen menu.

As data were stored locally on the device, the Rams Have Heart app operated offline, without requiring a continuous cellular or internet connection. Data were stored using a 128-bit advanced encryption standard algorithm with no PII. Whenever Wi-Fi internet access was available, they could be uploaded to a central, secure server to reduce the use of the participants’ cellular data plans. They were transferred using the secure https protocol and stored in a secure SQL server database, which was only accessible to authorized persons via user ID and password authentication.

## Results

### Study Cohort

Upon study entry, participants completed a paper questionnaire to collect demographic data. Of the total 109, 104(95%) completed the questionnaire; 83 (76%) were women, and 26 (24%) were men (Table 1). Most were 18 years old (76/109, 69.7%), the common age for first-year college students. Over half (56/109, 51.3%) had a normal BMI (18.5-24.9 kg/m^2^; Table 1).

**Table 1:** Demographic characteristics of participants (N=109).

Demographics^a^		Values, n (%)
**Age (years)**		
	17	8 (7.3)
	18	76 (69.7)
	19	13 (11.9)
	20	8 (7.3)
	≥21	4 (3.6)
**Sex**		
	Male	26 (23.8)
	Female	83 (76.1)
**BMI (kg/m^2^)**		
	Underweight (<18.5)	3 (2.7)
	Normal (18.5-24.9)	56 (51.3)
	Overweight (25.0-29.9)	28 (25.6)
	Obese (≥30.0)	22 (20.1)

^a^Data represent cohort 2 and cohort 3 combined. Data were collected from separate questionnaires external to the mobile app.

The demographic composition of the entire participant population and those in the intervention group did not differ significantly (Table 2). Of the 55 participants in the intervention group, 27 (49%) in cohort 2 and 25 (45%) in cohort 3 used the Rams Have Heart app at least once.

**Table 2:** Demographic characteristics of the intervention group (N=55).

Demographics^a^		Values, n (%)	*P* value^b^
**Age (years)**			
	17	4 (7)	.51
	18	37 (67)	.62
	19	4 (7)	.84
	20	7 (13)	.15
	≥21	3 (5)	.31
**Sex**			
	Male	19 (35)	.08
	Female	36 (65)	.92
**BMI (kg/m^2^)**			
	Underweight (<18.5)	2 (4)	.38
	Normal (18.5-24.9)	29 (53)	.43
	Overweight (25.0-29.9)	16 (29)	.32
	Obese (≥30.0)	8 (15)	.82

^a^Data represent cohort 2 intervention and cohort 3 intervention combined. Data were collected from separate questionnaires external to the mobile app and are representative of the original pool of participants who provided diary data.

^b^*P* value refers to the significance of the difference between the entire population of cohorts and the intervention population (Table 1).

### Use of Information Slides

For each module that requested manual user entry, participants could opt to view a brief introductory presentation, explaining, for example, what a serving of fruit looks like or the different levels of physical activity (ie, moderate vs vigorous). Of the 52 participants who used the app more than once, about 26 (50%) watched it the first time they visited the fruit and vegetable intake module, and 22 (42%) watched the physical activity module (Table 3). They could also watch it again whenever they visited the module. Throughout the entire study period, 37 participants (71%) watched the fruit and vegetable intake introductory presentation, and 34 (65%) watched the physical activity introductory presentation at least once (Table 3).

**Table 3:** Viewership of information slides (N=52).

Module^a^		Values, n (%)	
**Fruit and vegetable intake**			
	Watched before the first entry		26 (50)
	Watched at least once		37 (71)
**Physical activity**			
	Watched before the first entry		22 (42)
	Watched at least once		34 (65)

^a^Data were calculated based on participant ID and a true or false record of viewing the module in the dataset.

### App Use Over the Study Period

**Figure 7:**
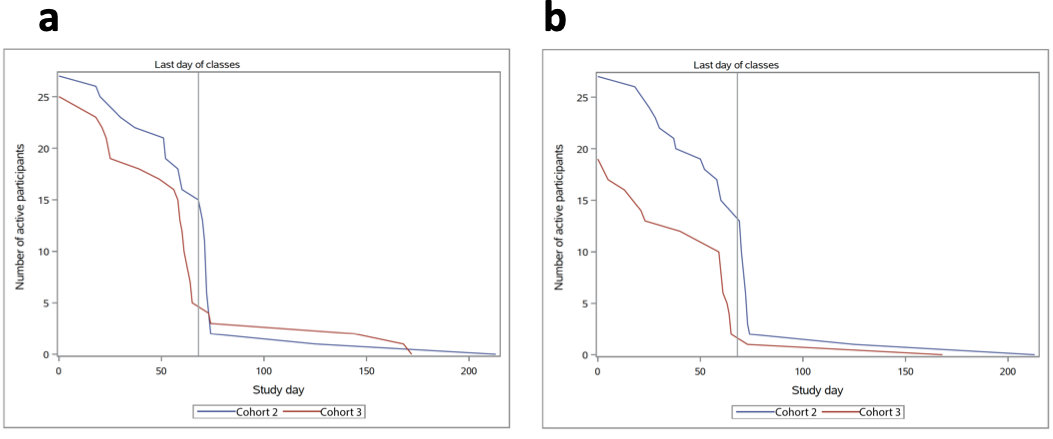
The number of active participants recording diary entries over the course of the study (a) active participants providing activity diary entries and (b) active participants providing diary entries of fruit and vegetables. The study day of drop-off was determined by the latest date of data entry for each module.

The study ran from late September 2017 to spring 2018 for cohort 2 and September 2018 to spring 2019 for cohort 3, with the course conducted in the fall semester and follow-up data collection in the spring. Both cohorts received frequent in-class reminders to adhere to the data collection; they were encouraged to upload data during class and offered an incentive after the fall 2017 semester ended. Cohort 2 participants gradually tapered their use of the app until around day 68 when 78% stopped, corresponding to the final exam week. Cohort 3 also displayed a sharp drop in the days just before the final exam week; participants recording fruit and vegetable diary entries fell from 16 to 5, and those recording their physical activity from 10 to 2 (Figure 7). App use did not increase afterward.

**Figure 8:**
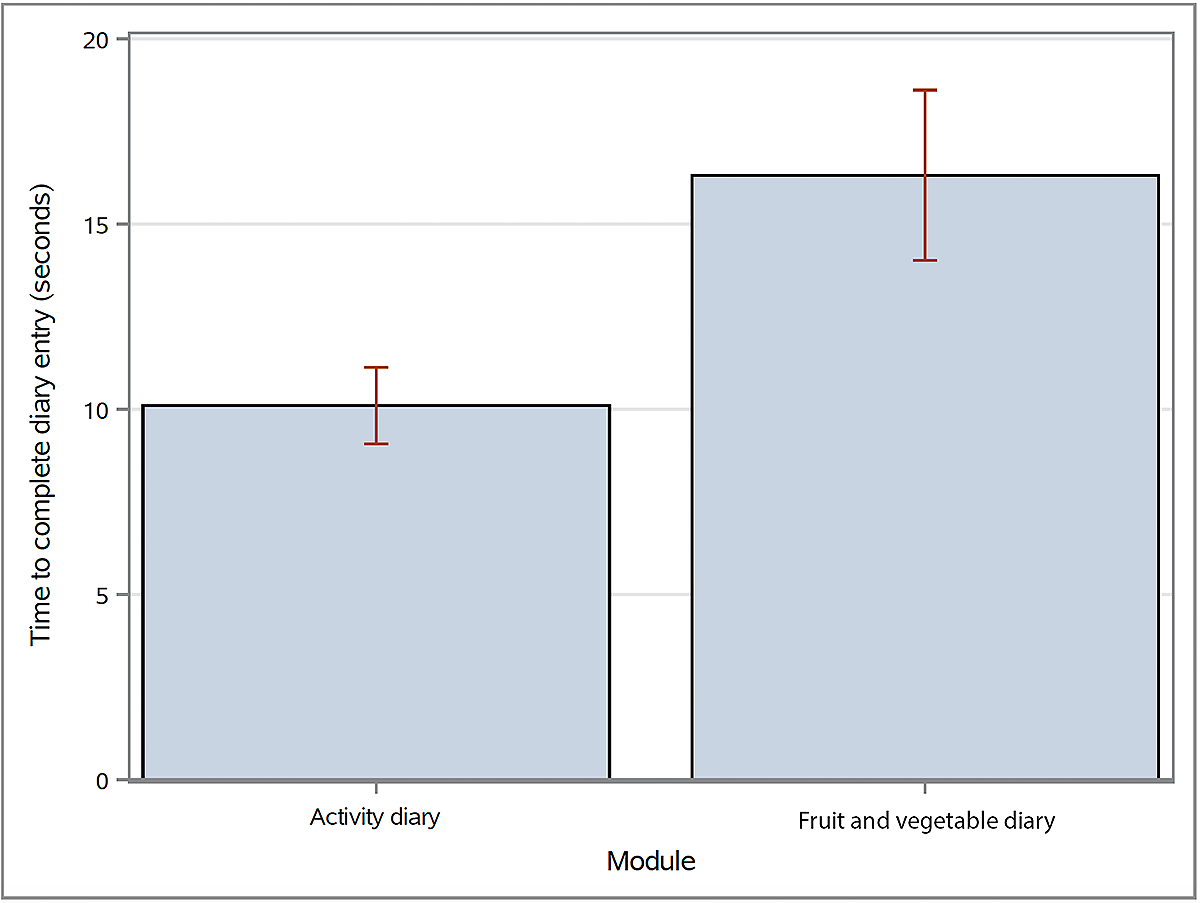
Average time participants took to enter a diary entry. Error bars represent Upper Confidence Level of the Mean (UCLM) and Lower Confidence Level of the Mean (LCLM). The average time was calculated by pooling all entries across all participants.

**Figure 9:**
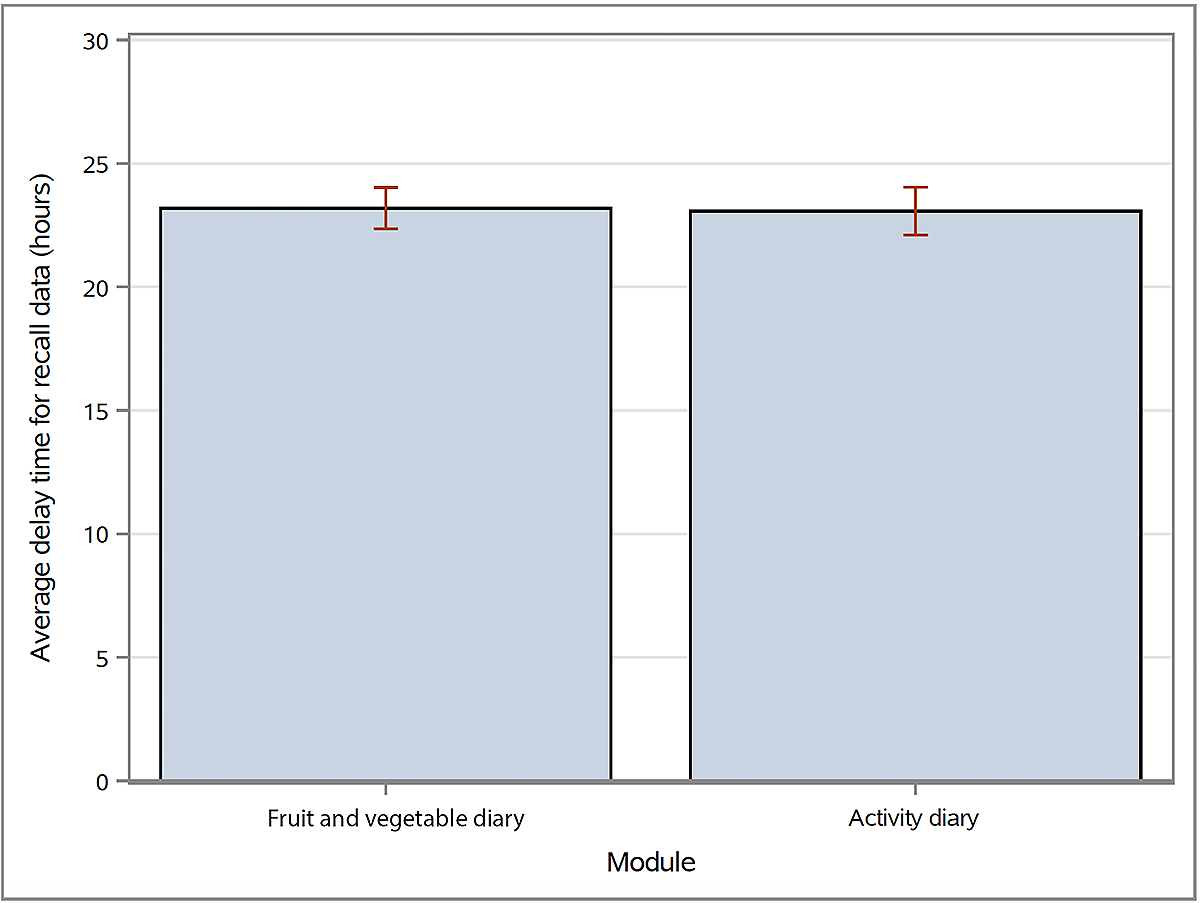
Average time participants took to enter a diary entry after the actual diary date. Error bars represent UCLM and LCLM. The average time was calculated by pooling all entries across all participants.

On average, the time participants took to record their fruit and vegetable intake was 16.3 seconds, and 10.1 seconds for activity (Figure 8). On average, they entered their recall data a full day late (Figure 9). Most entered their data for both diaries in the afternoon (1-3 PM). This trend was similar for recall data (Tables 4 and 5).

**Table 4:** Time of day data were recorded.

Time point	Fruit and vegetable intake n (%)^a^, N=1968	Activity n (%)^a^, N=1449
Midnight to 1 AM	114 (5.79)	120 (8.28)
1 AM to 2 AM	83 (4.22)	62 (4.28)
2 AM to 3 AM	120 (6.10)	95 (6.56)
3 AM to 4 AM	81 (4.12)	52 (3.59)
4 AM to 5 AM	66 (3.35)	56 (3.86)
5 AM to 6 AM	55 (2.79)	41 (2.83)
6 AM to 7 AM	19 (0.97)	17 (1.17)
7 AM to 8 AM	20 (1.02)	12 (0.83)
8 AM to 9 AM	5 (0.25)	3 (0.21)
9 AM to 10 AM	0 (0.00)	1 (0.07)
10 AM to 11 AM	1 (0.05)	1 (0.07)
11 AM to noon	11 (0.56)	10 (0.69)
Noon to 1 PM	42 (2.13)	30 (2.07)
1 PM to 2 PM	293 (14.89)	165 (11.39)
2 PM to 3 PM	269 (13.67)	208 (14.35)
3 PM to 4 PM	150 (7.62)	88 (6.07)
4 PM to 5 PM	67 (3.40)	58 (4.00)
5 PM to 6 PM	55 (2.79)	35 (2.42)
6 PM to 7 PM	86 (4.37)	60 (4.14)
7 PM to 8 PM	96 (4.88)	79 (5.45)
8 PM to 9 PM	69 (3.51)	56 (3.86)
9 PM to 10 PM	87 (4.42)	61 (4.21)
10 PM to 11 PM	108 (5.49)	81 (5.59)
11 PM to midnight	71 (3.61)	58 (4.00)

^a^Percentages indicate the proportion of entries recorded for the time period indicated. Data indicate all entries.

**Table 5:** Time of night data were recorded.

Time point	Fruit and vegetable intake (%)^a^ (N=1024)	Activity (%)^a^ (N=801)
Midnight to 1 AM	43 (4.20)	53 (6.62)
1 AM to 2 AM	39 (3.81)	30 (3.75)
2 AM to 3 AM	42 (4.10)	37 (4.62)
3 AM to 4 AM	41 (4.00)	24 (3.00)
4 AM to 5 AM	38 (3.71)	36 (4.49)
5 AM to 6 AM	35 (3.42)	29 (3.62)
6 AM to 7 AM	14 (1.37)	12 (1.50)
7 AM to 8 AM	13 (1.27)	9 (1.12)
8 AM to 9 AM	3 (0.29)	2 (0.25)
9 AM to 10 AM	0 (0.00)	1 (0.12)
10 AM to 11 AM	1 (0.10)	1 (0.12)
11 AM to noon	7 (0.68)	6 (0.75)
Noon to 1 PM	28 (2.73)	21 (2.62)
1 PM to 2 PM	178 (17.38)	111 (13.86)
2 PM to 3 PM	131 (12.79)	103 (12.86)
3 PM to 4 PM	81 (7.91)	58 (7.24)
4 PM to 5 PM	37 (2.61)	30 (3.75)
5 PM to 6 PM	33 (3.22)	22 (2.75)
6 PM to 7 PM	43 (4.20)	33 (4.12)
7 PM to 8 PM	48 (4.69)	44 (5.49)
8 PM to 9 PM	35 (3.42)	32 (4.00)
9 PM to 10 PM	47 (4.59)	35 (4.37)
10 PM to 11 PM	49 (4.79)	43 (5.37)
11 PM to midnight	38 (3.71)	29 (3.62)

^a^Percentages indicate the proportion of entries recorded for the time period indicated. Data indicate only entries entered as recall data.

### Healthy Behavior Adaptation

**Figure 10:**
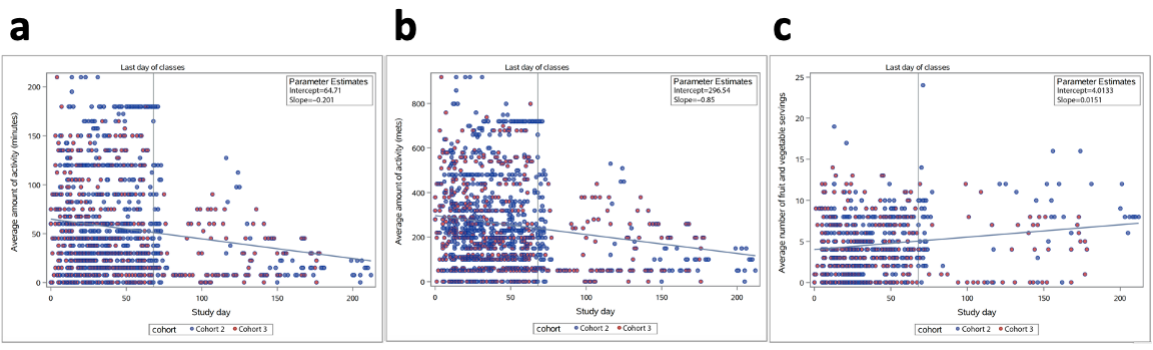
Diary entry values recorded over the study period: (a) activity data (in minutes) over time; (b) activity data (in METS) over time; (c) fruit and vegetable servings over time. METS: metabolic estimates.

Using the data entered on fruit and vegetable intake and activity level, we tracked individual and general trends over the study period. Note that the *n* of data (the total number of participants) was not constant and might skew the analysis of the results. Regression analysis showed that fruit and vegetable intake, as well as minutes and metabolic equivalents (METS) of activity (Figure 10) were all *P*<.001, indicating a positive correlation between days into the study and the measure studied. Activity levels in both minutes and METS decreased over time (Figure 10). Fruit and vegetable intake trended slightly upward as the study progressed (Figure 10).

**Figure 11:**
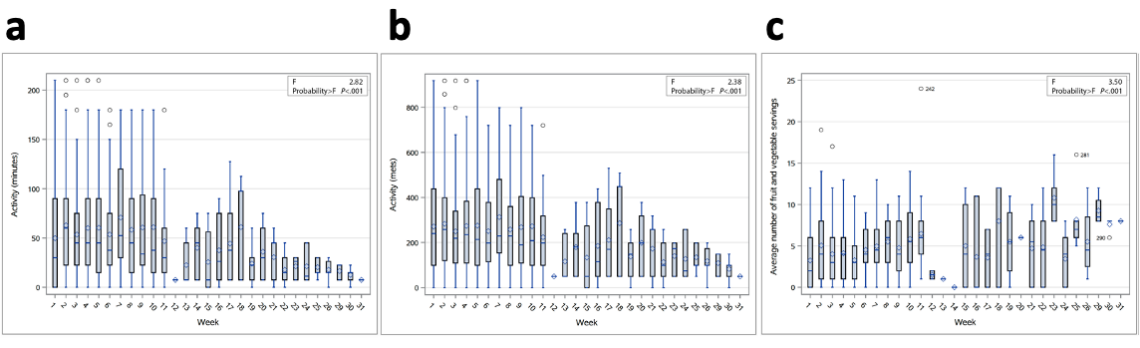
Analysis of variance of diary entries grouped per study week: (a) activity data (in minutes) over time; (b) activity data (in METS) over time; (c) fruit and vegetable servings over time. METS: metabolic estimates.

We ran an analysis of variance (ANOVA) using the Tukey two-tailed *t* test to determine whether the pooled data for cohorts 2 and 3 changed from week to week (Figure 11). The only significant finding (*P*=.03) occurred between weeks 1 and 2, with an average increase of 1.8 fruit and vegetable servings (average consumption for week 1=3.24, SD 3.50 and week 2=5.05, SD 4.15), but we found no significant change when comparing week 1 to week 3 (Table 6). Throughout the course of the study, average fruit and vegetable servings and minutes and METS of activity fluctuated (Figure 8).

**Table 6:** Trend of fruit and vegetable and activity results over time.

Study week^a^	Fruit and vegetable	*P* value	Activity (min)	*P* value	Activity (metabolic equivalents)	*P* value
2 and 1	1.8089	.03	13.074	.94	12.06	>.99
3 and 2	−1.0330	.97	−9.476	>.99	−33.44	>.99
4 and 3	0.1032	>.99	6.675	>.99	24.01	>.99
5 and 4	−0.8430	>.99	0.188	>.99	1.38	>.99
6 and 5	1.2351	.95	−6.796	>.99	−24.25	>.99
7 and 6	0.4428	>.99	17.207	.63	62.44	.84
8 and 7	0.5558	>.99	−12.434	.98	−55.25	.95
9 and 8	−0.7438	>.99	2.348	>.99	10.20	>.99
10 and 9	1.0697	>.99	0.315	>.99	4.21	>.99
11 and 10	0.6354	>.99	−14.137	>.99	−48.68	>.99
12 and 11	−4.9688	.98	−39.324	>.99	−174.16	>.99
13 and 12	−0.5000	>.99	15.000	>.99	66.50	>.99
14 and 13	−1.0000	>.99	17.500	>.99	63.50	>.99
15 and 14	5.0000	>.99	−14.375	>.99	−46.13	>.99
16 and 15	−1.3333	>.99	11.875	>.99	51.73	>.99
17 and 16	−0.0952	>.99	6.818	>.99	25.85	>.99
18 and 17	4.4286	.99	16.753	>.99	75.12	>.99
19 and 18	−2.5000	>.99	−37.071	>.99	−148.47	>.99
20 and 19	0.5000	>.99	12.000	>.99	61.00	>.99
21 and 20	−1.3000	>.99	−5.500	>.99	−25.90	>.99
22 and 21	0.1333	>.99	−12.773	>.99	−59.25	>.99
23 and 22	5.9667	.50	3.523	>.99	26.80	>.99
24 and 23	−7.3714	.07	0.000	>.99	−13.00	>.99
25 and 24	4.7381	.80	−0.893	>.99	7.11	>.99
26 and 25	−2.6667	>.99	−2.545	>.99	−16.79	>.99
27 and 26	—^b^	—	—	—	—	—
28 and 27	—	—	—	—	—	—
29 and 28	—	—	—	—	—	—
30 and 29	−1.6500	>.99	−115.217	>.99	−26.40	>.99
31 and 30	0.4000	>.99	−5.000	>.99	−33.00	>.99
31 and 1	4.7624	>.99	−42.361	>.99	−221.49	>.99

^a^Numbers represent the difference between means calculated using ANOVA with the Tukey studentized range (honestly significant difference) test, pooling data from cohort 2 and cohort 3.

^b^Data not available.

## Discussion

With the rise of mobile phone technology, several apps have been developed to tackle health problems. Many enable self-monitoring to achieve health-related goals and are promoted by medical practitioners to improve patient health [21,22,26]. Although Rams Have Heart featured modules similar to those used by other health-related mobile apps—for example, one counting daily fruit and vegetable servings—it was designed with a research question in mind. We did not assess whether participants were using any other apps for dietary or exercise improvement but based on the low adherence to our protocol, even as part of a college course, we deemed it unlikely.

Rams Have Heart aimed to monitor the adaptation of concepts from the CVD-oriented college course, not to actively support behavioral change. The app might have done more to support participant adherence, but the larger objective was to design a stand-alone course that would improve the cardiovascular health of students with or without this or any other app. Therefore, the app’s functionality is centered on its ability to collect data for further analysis of its usability and methods for improvement but, more importantly, achievement of course goals.

Studies of participant retention have suggested using mobile devices to collect data rather than burdening participants with phone calls or in-person appointments [27]. Flexibility is a key to promoting user engagement but can also lead to forgetfulness [28]. After the cohort 1 pilot study, increasing in-class reminders to use the app and adding incentives resulted in the longer adherence found in cohorts 2 and 3. Retention methods specific to the cohort are sometimes necessary in longitudinal studies [29]. In years 2 and 3, providing in-class reminders and time to upload data increased participant retention and the number of entries. Studies reviewing health research using mobile apps have shown that the average retention period is around 25.62 (SD 18.41) weeks, and participants who stick with the app over the long term typically have a strong incentive, such as a serious health problem [23]. Programmatic incentives include virtual rewards, such as accomplishment badges [30] and the ability to share on social media accounts [31-33], as well as more tangible rewards [34]. In spring 2018, our study introduced a monetary incentive: an Amazon gift card to the student who had the highest number of diary entries at the end of each month for the period after the course ended to the 6-month follow-up. However, only 1 student continued to provide entries. Introducing the incentive late in the process was not very effective.

As part of the diary modules, students could first watch informational slides. Although most skipped them when they first recorded a diary entry, 71% watched the fruit and vegetable servings presentation at least once, and 65% watched the activity presentation at least once. The physical activity module did not require much explanation, asking merely for activity time in minutes. College students are less likely to understand serving sizes and numbers of servings [35-37], which may account for the higher viewership of this presentation.

On average, fruit and vegetable serving entries took 5 seconds longer than the activity diary entries (Figure 8), possibly because they involved more rows—fruits and/or vegetables consumed at breakfast, lunch, dinner, and as snacks—or 8 rows as compared with only 3 rows for activity (Figure 3). However, this difference did not affect the average time of recall data entry (Figure 9) nor account for the difference in the number of participants inputting diary entries over the study period for either the fruit and vegetable serving diary or the activity diary (Figure 7).

All of the diary data were acquired using the app either in near real time or the app-supported recall within 1 or 2 days. No data were acquired via nondigital means, such as a paper diary. Although the student participants often failed to make daily diary entries, no attempt was made to estimate and *fill in* missing entries.

We saw no significant improvement in average fruit and vegetable servings or activity in minutes or METS when comparing the last week of the study to the first week (Table 6). Mobile apps with self-reported health assessments (eg, calorie counting and food diaries) rely on user input to provide useful feedback on improvements or when to seek professional help. When these measures are used for research data collection, accuracy and a large data pool are required [38]. In this study, most participants did not continue to record their food intake and activity levels because other life events, such as the end of the semester and the start of final exams, took precedence [39]. Other retention methods, such as text messages, might have improved the frequency of entries and reduced the number of dropouts.

### Conclusions

Rams Have Heart was developed to enhance fruit and vegetable intake and physical activity for a student demographic susceptible to obesity, heart disease, and type 2 diabetes. As part of the study, we conducted an analysis of the functionality and usage of a health-related mobile app. Although Rams Have Heart provides privacy and flexibility for user participation in a research study, it did not improve participant retention or user outcomes. This finding calls for further evaluation to determine more effective retention methods.

## Abbreviations

AAs: African Americans
AIR: Adobe integrated runtime
ANOVA: analysis of variance
BP: blood pressure
CVD: cardiovascular disease
HBCU: historically black college and university
ID: identifier
IPAQ: International Physical Activity Questionnaire
IRB: institutional review board
METS: metabolic equivalents
mHealth: mobile health
PHIT: Personal Health Informatics and Intervention Toolkit
PII: personally identifiable information
PIN: Personal Identification Number
SQL: Structured Query Language
